# Clinical and trichoscopic features of early congenital syphilis: a single-center cross-sectional study

**DOI:** 10.3389/fmed.2026.1748553

**Published:** 2026-02-18

**Authors:** Cuauhtli Emmanuel Arambul-Carrillo, Luis Enrique Sánchez-Dueñas, Rocío Concepción Albores-Arguijo, Yocelin Nallely Lozano-Figueroa, María Guadalupe Castro-Mosqueda, Jessica Aranda-Mendoza

**Affiliations:** 1 Antiguo Hospital Civil de Guadalajara-Fray Antonio Alcalde, Pediatric Dermatology Department, University of Guadalajara, Guadalajara, Mexico; 2 Dermatologic Institut of Jalisco "Dr. José Barba Rubio", Department of Dermatology, University of Guadalajara, Guadalajara, Mexico; 3 Adjunct Professor in the Specialty of Pediatric Dermatology, University of Guadalajara, Guadalajara, Mexico

**Keywords:** alopecia, congenital syphilis, syphilis, syphilitic alopecia, trichoscopy

## Abstract

**Introduction:**

Early congenital syphilis (ECS) remains an emerging public health problem in Latin America. Syphilitic alopecia (SA) is a rare and underreported manifestation in neonates, and its trichoscopic characteristics have not yet been systematically described.

**Methods:**

We have conducted a 1-year cross-sectional analytical study involving 30 neonates diagnosed with ECS, classified into clinical–serological scenarios 1 and 2 according to the Centers for Disease Control and Prevention (CDC) criteria, who were hospitalized at a tertiary care center in Guadalajara, Mexico. Sociodemographic, clinical, and serological data were collected. The presence of SA and associated trichoscopic findings was recorded. Neonates were stratified according to non-treponemal titers into two groups [<1:128 vs. ≥1:128 and/or the prozone phenomenon (PP)]. Associations were evaluated using the chi-squared test or Fisher’s exact test, as appropriate. Logistic regression models were used to examine the association between trichoscopic characteristics and elevated neonatal Venereal Disease Research Laboratory (VDRL) titers.

**Results:**

Thirty neonates were included, yielding an ECS prevalence of 3.05%. The vast majority of patients were male (76.7%) and were classified as CDC scenario 2 (70%). SA was identified in 23.3% of cases and was significantly associated with VDRL titers ≥1:128 or the presence of a prozone phenomenon (*p* = 0.006; odds ratio [OR]: 11.9; 95% confidence interval [CI]: 1.67–84.50). The most frequent trichoscopic abnormalities were exclamation mark hairs, coudability hairs, broken hairs, zig-zag/angulated hairs and Pohl–Pinkus constrictions. Among these abnormalities, exclamation mark and coudability hairs have shown the strongest associations with elevated neonatal VDRL titers.

**Conclusion:**

Trichoscopy enabled the identification of specific hair shaft abnormalities associated with increased serological activity, supporting its value as a complementary, non-invasive diagnostic tool in pediatric dermatology for evaluating neonates with ECS.

## 1 Introduction

Congenital syphilis (CS) is an infectious disease caused by *Treponema pallidum* and transmitted primarily via the placenta, although peripartum transmission may also occur through direct contact with active maternal lesions during delivery (1, 2). Clinically, CS is classified as early when manifestations occur within the first 2 years of life and later when they present thereafter.

In contrast, acquired syphilis develops beyond the neonatal period and classically progresses through the primary, secondary, latent, and tertiary stages, with neurosyphilis representing central nervous system involvement (1, 3).

Despite being entirely preventable and treatable, CS has experienced a concerning resurgence. The United States reported a 500% increase in cases between 2011 and 2020, according to the Centers for Disease Control and Prevention (CDC) (4). Similarly, in Mexico, the National Epidemiological Bulletin reported an incidence of 3.20 cases per 10,000 live births between 2019 and 2023 (5). Comparable trends across Latin America reflect persistent gaps in prenatal screening, maternal treatment, and access to adequate antenatal care (6).

Syphilis is widely recognized as the “great imitator” due to its capacity to affect virtually any organ system (4). In its congenital form, mucocutaneous manifestations are among the most common clinical findings (7). These manifestations include maculopapular or morbilliform eruptions, papulosquamous plaques with Biett’s collarette, acral desquamation (pemphigus syphiliticus), mucosal erosions, petechiae, erythema multiforme–like lesions, condyloma lata, and, less frequently, patchy or diffuse alopecia (4, 7). Nevertheless, up to two-thirds of affected neonates may be asymptomatic at birth or show only subtle clinical signs, which typically become evident within the first 6 weeks of life in nearly 70% of cases (1, 2).

Syphilitic alopecia (SA) is an uncommon but well-described manifestation, occurring in approximately 4% of adult patients with syphilis. McCarthy’s 1940 classification—still accepted in recent times—distinguishes a symptomatic form associated with papulosquamous scalp lesions, from an essential form characterized by non-scarring hair loss in the absence of visible luetic lesions, typically presenting in moth-eaten, diffuse, or mixed patterns (8). Recent histopathological and molecular studies reinforce the epitheliotropism of *T. pallidum* and a specific immune-mediated follicular response, supporting the biological plausibility of hair involvement (8–10).

Trichoscopy, introduced by Rudnicka et al. in 2006, enables magnified visualization of scalp and hair features—including follicular openings, perifollicular structures, hair shafts, and vascular patterns—using handheld or digital dermatoscopes (11, 12). This non-invasive technique has become an essential tool for differentiating hair and scalp disorders and for reducing the need for biopsy (12, 13). Standardized frameworks classify trichoscopic findings into five major groups: follicular, perifollicular, scalp surface, hair distribution patterns, and hair shaft abnormalities (13–15). Although congenital hair shaft dysplasias are not explicitly included within these classification systems, trichoscopy has increasingly been recognized as a valuable diagnostic tool in neonates and infants with genodermatoses and as hereditary forms of hypotrichosis, as it enables early, non-invasive assessment of hair and scalp abnormalities in this population (16–19).

In adults, the trichoscopic characteristics of SA were outlined by Ye et al. (20), followed by Piraccini et al. (21), who used trichoscopy as a diagnostic tool in cases of secondary syphilis, emphasizing that SA may represent the only manifestation of secondary disease (21). More recent reviews have detailed trichoscopic findings associated with SA, highlighting alterations in hair shaft morphology, follicular and perifollicular structures, scalp surface characteristics, and hair distribution patterns (22, 23).

Diagnosing CS during the neonatal period remains particularly complex, as maternal antibodies may interfere with serological interpretation, and many infants are asymptomatic. The CDC guidelines recommend a comprehensive diagnostic approach that integrates maternal history, clinical findings, and quantitative non-treponemal testing using Venereal Disease Research Laboratory (VDRL) or Rapid Plasma Reagin (RPR) assays (24–26). Based on these parameters, neonates are classified according to the Centers for Disease Control and Prevention (CDC) guidelines into four diagnostic clinical scenarios reflecting the probability of infection: confirmed or highly probable CS (scenario 1), possible CS (scenario 2), less-likely CS (scenario 3), and unlikely CS (scenario 4) (1, 25, 26).

However, diagnosis during the neonatal period remains challenging. Non-treponemal tests may yield false-negative results due to the prozone phenomenon (PP) or false-positive results in other conditions, such as antiphospholipid syndrome, while treponemal assays are not routinely recommended as maternal antibodies may confuse interpretation (7, 25, 26). These limitations highlight the need for complementary diagnostic tools to enhance early recognition. In this context, trichoscopy may be a valuable aid for the early identification of CS. Therefore, the objective of this study is to (i) describe trichoscopic findings in neonates with early congenital syphilis (ECS), (ii) assess their association with clinical and serologic parameters, and (iii) explore their possible diagnostic role in CDC scenarios 1 and 2.

## 2 Materials and methods

### 2.1 Study design, settings, and participants

This single-center, cross-sectional analytical study included neonates diagnosed with ECS who were born or admitted to the Neonatal Intensive Care Units (NICUs) of the “Fray Antonio Alcalde” Civil Hospital of Guadalajara (HCFAA), a tertiary referral center in Guadalajara, Mexico. Data were collected between November 2024 and October 2025. The inclusion criteria were as follows: (1) neonates <29 days old; and (2) diagnosis of ECS classified as scenario 1 or 2 according to CDC criteria. Scenario 1 corresponds to confirmed or highly probable CS (clinical abnormalities or neonatal non-treponemal titers ≥4-fold maternal levels). In contrast, scenario 2 includes possible CS with regular examination and titers ≤4-fold maternal levels in the setting of absent, inadequate, or late maternal treatment. The exclusion criteria were as follows: (1) congenital genodermatoses or hypotrichosis; (2) neonates born to mothers with antiphospholipid syndrome; (3) poor-quality images; and (4) failure to fulfill CDC scenario 1 or 2 criteria.

### 2.2 Study variables and data sources

For each participant, the following data were collected: neonatal variables (sex, gestational age, birth weight, and parity and delivery modes), maternal variables (age, number of prenatal visits, comorbidities, and drug use), and clinical–serologic variables (including neonatal and maternal semi-quantitative VDRL titer at diagnosis, CDC ECS scenarios, the maternal stage of acquired syphilis, neonatal mucocutaneous findings, and alopecia pattern). Neonatal trichoscopic alterations were recorded according to the nomenclature proposed by Kinoshita-Ise et al. (14), encompassing follicular, perifollicular, scalp-surface, hair-distribution, and hair-shaft findings. All examinations were performed using a Heine Delta One® dermatoscope (HEINE Optotechnik GmbH & Co. KG, Gilching, Germany) at 10 × magnification. A trichoscopic evaluation was conducted in a systematic and standardized manner, following a predefined protocol including a dry examination followed by a wet examination with a water-based gel. The eyebrows and the frontal, temporal, parietal, and occipital regions of the scalp were evaluated sequentially, paying particular attention to clinically alopecic areas.

Trichoscopic images were analyzed independently by two researchers—a pediatric dermatologist and a dermatologist with specific training in trichology—who were unaware of the neonatal serological titers in order to reduce the risk of observer bias.

No formal interobserver agreement analysis was performed due to the limited sample size and exploratory nature of the study. Discrepancies in the interpretation of the images were resolved by consensus following a joint review of the images, being acknowledged as a methodological limitation.

### 2.3 Statistical analysis

The prevalence of ECS was estimated, and its distribution was described according to neonatal sociodemographic, clinical–serologic, and trichoscopic variables. Data normality was assessed within each group using the Shapiro–Wilk test.

For comparative analyses, neonates were stratified according to semi-quantitative VDRL titers. Associations between VDRL titers and clinical and trichoscopic findings were evaluated at predefined titer levels (1:8, 1:16, 1:32, 1:64, 1:128, and 1:256). A statistically significant association was identified at a cut-off value of 1:128, allowing two groups to be defined: Group 1 (<1:128) and Group 2 (≥1:128 or presence of a PP).

Statistical analyses were performed using Jamovi software (version 2.3.18). Categorical variables were analyzed using the chi-squared test and Fisher’s exact test, as appropriate, and a *p*-value of <0.05 was considered statistically significant. A logistic regression analysis was conducted to evaluate the associations between trichoscopic findings and higher neonatal VDRL titers (Group 2), and results were expressed as odds ratios (ORs) with corresponding 95% confidence intervals (95% CIs). No *post-hoc* power analysis was performed due to the exploratory nature of the study and the limited sample size.

## 3 Results

### 3.1 Prevalence, sociodemographic, and serological features of early congenital syphilis

During the study period, 32 neonates with ECS were identified. Two were excluded—one with caudal regression syndrome and one due to inconclusive trichoscopic images—resulting in a final total of 30 neonates, corresponding to a prevalence 3.05% among NICU admissions at HCFAA.

The most affected infants were male (76.7%; *n* = 23), those born at ≥37 weeks of gestation [GW; full-term; 70%; *n* = 21], and delivered vaginally (50%; *n* = 15), with a mean gestational age of 38.1 [standard deviation (SD) ± 2.44] weeks and a mean birth weight of 2,822 g (SD ± 644 g). Mothers had a mean age of 25.2 (SD ± 6.61) years and attended an average of 2.7 (SD ± 2.59) prenatal visits; notably, 30% (*n* = 9) had no prenatal care. Maternal drug use during pregnancy was reported in 53.3% (*n* = 16), primarily involving methamphetamine derivatives, 43.4% (*n* = 13).

The majority of the neonates (70%) exhibited VDRL titers <1:128 (*n* = 21), with two cases (6.6%) demonstrating the PP. The mean neonatal VDRL titers were 1:45 (1:2–1:2048). According to CDC criteria, 70% (*n* = 21) were classified as scenario 2, whereas 30% (*n* = 9) were classified as scenario 1. Among scenario 1 cases, 66.7% (*n* = 6) presented VDRL titers ≥1:128, while 33.3% (*n* = 3) exhibited VDRL titers <1:128. The mean maternal VDRL titer was 1:187 (1:2–1:512), with 13.2% (*n* = 4) showing the PP. The most frequent maternal disease stage was latent syphilis (60%; *n* = 18), followed by secondary syphilis (33.4%; *n* = 10), and primary syphilis or neurosyphilis (6.6%; *n* = 2).

### 3.2 Clinical and trichoscopic findings in neonates

**Figure 1:**
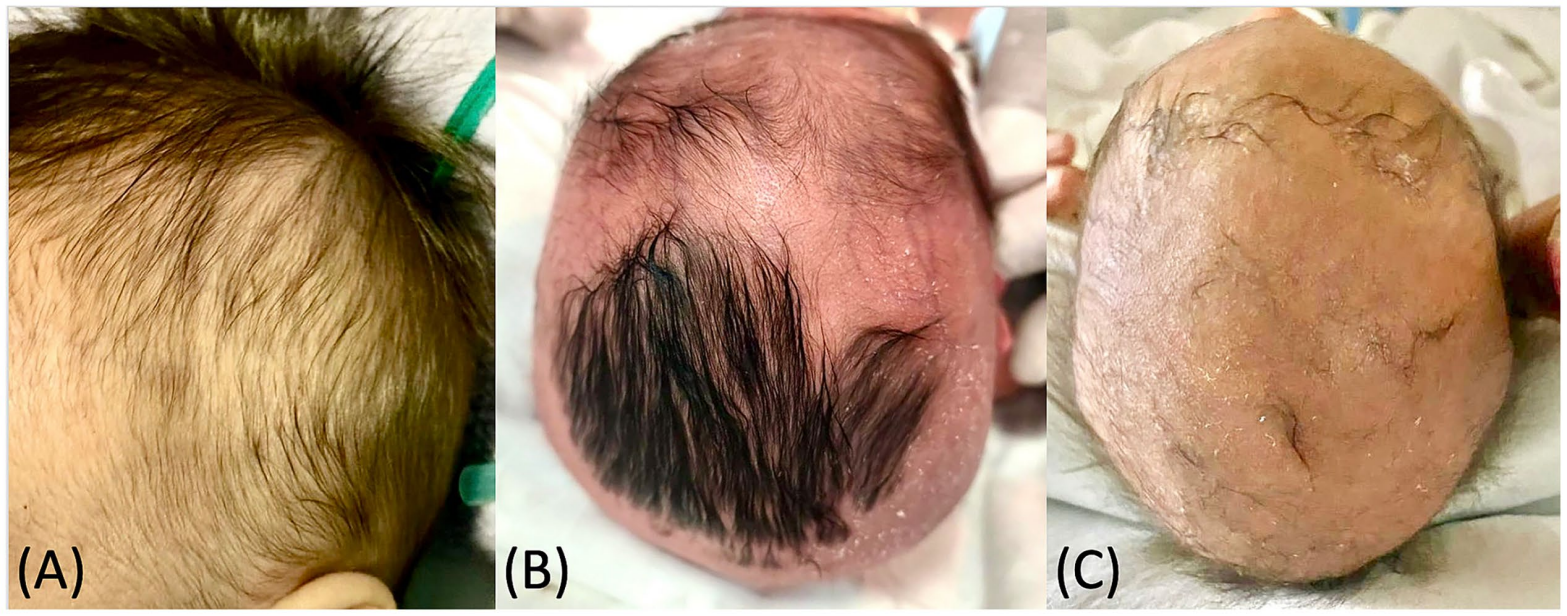
Clinical photographs of neonates with syphilitic alopecia: **(A)** neonate boy with small “moth-eaten” pattern patches; **(B)** neonate boy with patchy alopecia in a halo-ring pattern; **(C)** neonate boy with extensive patchy alopecia involving almost the entire scalp.

Clinically, alopecia occurred in 23.3% (*n* = 7) of neonates, predominantly 83.3% (*n* = 6) in males. Small moth-eaten pattern patches predominated 71.4% (*n* = 5) (Figure 1A), while two cases (28.6%) showed larger alopecic plaques—one with a halo-ring pattern (Figure 1B), and another involving the interparietal and vertex regions (Figure 1C). Mucocutaneous alterations were observed in 60% (*n* = 18) of cases, including palmoplantar desquamation, maculopapular eruptions, and Biett’s collarette. When comparing clinical findings between groups, alopecia showed a significant association with elevated VDRL titers (*p* = 0.006; OR: 11.9, 95% CI: 1.67–84.50), whereas mucocutaneous alterations did not show a significant association (*p* = 0.193; OR: 3.18, 95% CI: 0.53–19.10). Clinical findings are summarized by group stratification in Table 1. It is important to note that none of the sociodemographic or maternal variables were significantly associated with neonatal VDRL titers in the regression model.

**Table 1:** Clinical findings according to the subdivision of groups based on neonatal serum VDRL titers.

Clinical variables	Total *N* = 30 (%, *n*/30)	Group 1 (VDRL <1:128) 70%, 21 (%, *n*)	Group 2 (VDRL ≥1:128 or PP) 30%, 9 (%, *n*)	*p*-value	OR (95% CI)
Presence of mucocutaneous alterations	60.0%, 18/30	36.7%, 11/30	23.3%, 7/30	0.193	3.18 (0.53–19.10)
Absence of mucocutaneous alterations	40.0%, 12/30	33.3%, 9/30	6.7%, 3/30		
Presence of alopecia	23.3%, 7/30	6.7%, 2/30	16.6%, 5/30	**0.006**	**11.9 (1.67–84.50)**
Absence of alopecia	76.7%, 23/30	63.3%, 19/30	13.3%, 4/30		

VDRL, The Venereal Disease Research Laboratory test titers; PP, prozone phenomenon; OR, odds ratio; CI, confidence interval.Bold values indicate statistically significant results.

**Figure 2:**
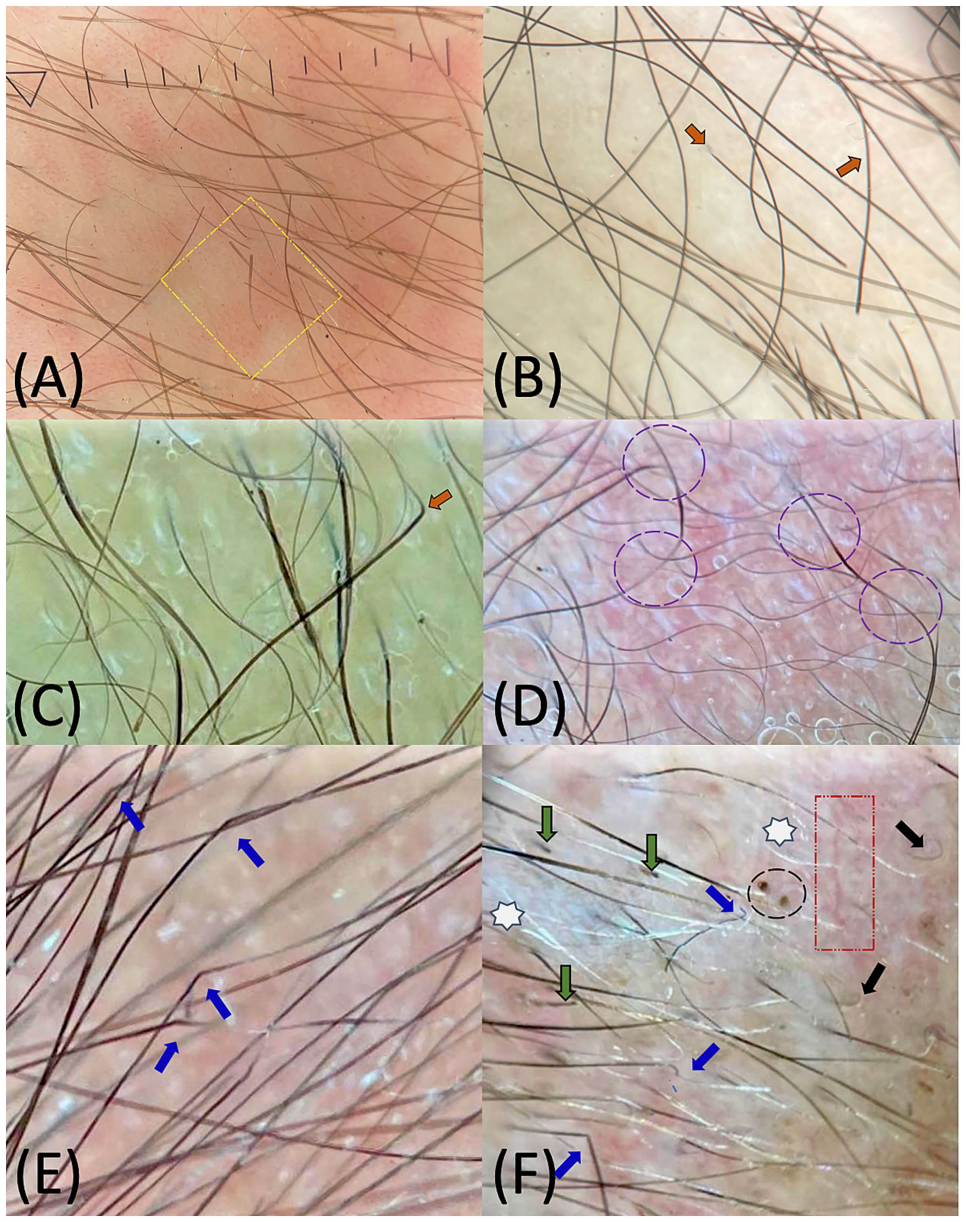
10 × non-polarized trichoscopy of the scalp in neonates with congenital syphilis: **(A)** Wet trichoscopy demonstrating exclamation-mark hairs (yellow square); **(B,C)** Wet trichoscopy showing coudability hairs (orange arrows); **(D)** Wet trichoscopy highlighting Pohl–Pinkus constrictions (purple circles); **(E)** Dry trichoscopy with zig-zag/angulated hairs (blue arrows); **(F)** Dry trichoscopy showing variability of trichoscopic findings, including zig-zag/angulated hairs (blue arrows), “pig-tail” hairs (black arrows), broken hairs (green arrows), black dots (black circle), arborizing vessels (red box), and leukotrichia (white stars).

Regarding trichoscopic findings, several alterations were significantly more frequent in Group 2, including exclamation-mark hairs (*p* < 0.001) (Figure 2A), coudability hairs (*p* = 0.005) (Figures 2B,C), Pohl–Pinkus constrictions (*p* = 0.025) (Figure 2D), zig-zag/angulated hairs (Figure 2E) (*p* = 0.046) and broken hairs (*p* = 0.035) (Figure 2F). Among these findings, exclamation-mark hairs (OR: 52.6, 95% CI: 2.44–1,130.0) and coudability hairs (OR: 23.2, 95% CI: 1.02–509.0) showed the strongest associations. All documented trichoscopic findings are summarized in Table 2.

**Table 2:** Trichoscopic findings documented in the subdivision of groups based on the neonatal serum VDRL titers.

Location findings	Trichoscopic findings variables	Total *N* = 30* (%, *n*)	Group 1 (VDRL <1:128) 70%, 21 (%, *n*)	Group 2 (VDRL ≥1:128 or PP) 30%, 9 (%, *n*)	*p*-value	OR (95% CI)
Follicular findings	Yellow dots	3.3%, 1	0%, 0	3.3%, 1	0.120	7.59 (0.28–205.00)
	White dots	3.3%, 1	0%, 0	3.3%, 1	0.120	7.59 (0.28–205.00)
	Red dots	6.6%, 2	3.3%, 1	3.3%, 1	0.523	2.50 (0.13–45.00)
Hair shaft findings	Pohl–Pinkus constrictions	6.6%, 2	0%, 0	6.6%, 2	**0.025**	14.30 (0.61–334.00)
	Coiled hair	10.0%, 3	10.0%, 3	0%, 0	0.232	10.00 (0.87–115.00)
	Coudability hair	10.0%, 3	0%, 0	10.0%, 3	**0.005**	**23.20 (1.02–509.00)**
	Broken hair	13.3%, 4	3.3%, 1	10.0%, 3	**0.035**	10.0 (0.87–115.00)
	Exclamation mark hair	16.6%, 5	0%, 0	16.6%, 5	**<0.001**	**52.60 (2.44–1130.00)**
	Zig-zag/angulated hair	30.0%, 9	13.3%, 4	16.6%, 5	**0.046**	5.31 (0.96–29.30)
	Pigtail hair	56.6%, 17	43.3%, 13	13.3%, 4	0.376	0.49 (0.10–2-40)
	Black dots	56.6%, 17	36.6%, 11	20.0%, 6	0.469	1.82 (0.35–9.27)
Perifollicular or interfollicular findings	Interfollicular scales	10.0%, 3	6.6%, 2	3.3%, 1	0.894	1.19 (0.09–15.0)
	Perifollicular scales	43.3%, 13	26.6%, 8	16.6%, 5	0.376	2.03 (0.41–9.89)
	Twisted red loops	3.3%, 1	0%, 0	3.3%, 1	0.120	7.59 (0.28–205.00)
	Arborizing red lines	63.3%, 19	43.3%, 13	20.0%, 6	0.840	1.23 (0.23–6.36)

VDRL, The Venereal Disease Research Laboratory test titers; PP, prozone phenomenon; OR, odds ratio; CI, confidence interval. * Trichoscopic alterations were observed in 16.6% (*n* = 5) of cases with at least one alteration, 36.7% (*n* = 11) with two alterations, and 46.7% (*n* = 14) with three or more alterations.Bold values indicate statistically significant results.

Other features, including yellow, white, or red dots, perifollicular and interfollicular scaling, twisted or arborizing vessels, showed no significant intergroup differences. Although leukotrichia was observed in two patients (Figure 2F), these findings did not meet the criteria for classification as true hair pigmentation disorders and were therefore excluded from the analysis of trichoscopic alterations.

## 4 Discussion

This analytical cross-sectional study described the clinical and trichoscopic manifestations of 30 neonates with early ECS and analyzed their association with neonatal VDRL titers according to CDC clinical–serologic scenarios 1 and 2. The prevalence observed (3.05%) was similar to other Latin American reports, though estimates vary widely across countries (2–69.3%), due to differing diagnostic criteria and prenatal screening coverage (6, 27). These results underscore that ECS remains a significant public health concern in the region despite established maternal screening and treatment programs.

Regarding the maternal profile, the majority of the mothers were young and had latent syphilis (60%), with a mean age of 25.2 years—consistent with previous Mexican data reporting a mean age of 23 years and a predominance of latent infection (92.7%) (28). Prenatal care was inadequate, with a mean of 2.7 prenatal visits and absence of follow-up in 30% of cases, likely contributing to persistent vertical transmission. High maternal VDRL titers (mean 1:187) reinforce their value as indirect markers of infectious activity and vertical transmission risk. Drug use during pregnancy, documented in 53.3% of cases, further underscores the need for integrated preventive strategies, including sexually transmitted disease screening and multidisciplinary care for substance use during pregnancy.

Among neonatal clinical–serologic variables, a male predominance (76.7%) was observed, contrasting with female predominance reported in other series (29, 30). Scenario 2 was the most frequent (70%), similar to the findings in a recent Mexican cohort (28). The mean neonatal VDRL titers (1:45) were elevated, though direct comparisons were limited, as the majority of studies report only maternal titers. Nevertheless, these values suggest active infection, as CDC scenario 1 classification requires neonatal titers at least 4-fold higher than maternal titers.

Trichoscopy use in the neonatal period has been scarcely documented. Neri et al. (2013) (31) reported that healthy neonates, with a mean birth weight of approximately 2,850 g, exhibit lower hair density and thinner shafts compared with adults, primarily in the frontal and temporal regions, correlating with gestational age and birth weight. They also observed vellus hair, mild scaling, and visible capillaries; among hair shaft abnormalities, only the presence of pigtail-shaped hairs was documented, with no other findings on the hair shaft or scalp (31). In our study (mean birth weight: 2,822 g), similar findings were observed and did not correlate with VDRL titers, suggesting physiological rather than disease-specific features (16, 31).

During the neonatal period, congenital hair shaft abnormalities within the spectrum of hair shaft dysplasias are primarily associated with genodermatoses and hereditary hypotrichosis. Trichorrhexis invaginata is a pathognomonic finding of Netherton syndrome, while other congenital hair shaft dysplasias have not been described in healthy neonates (16–19). Among acquired conditions, zig-zag hair has been described as a characteristic trichoscopic finding of *tinea capitis* caused by *Microsporum canis,* secondary to *ectotrix* parasitism, which causes fragility and fracture of the hair shaft (32).

Alopecia in the neonatal and pediatric period encompasses a wide range of congenital and acquired causes, including hair shaft abnormalities, inflammatory disorders, infections, and metabolic conditions (15, 33, 34). However, SA in the context of CS has rarely been considered within this age group (33, 34). Historically, reports of syphilitic alopecia in ECS have been sporadic and are summarized in Table 3 (3, 35–38). The first descriptions date back to Barlow (36), and Wechselberg and Schneider (35) subsequently documented a prevalence of approximately 3% in a German cohort. More recent case reports have described moth-eaten alopecia in neonates with ECS and elevated non-treponemal titers measured by the RPR test, including a titer of 1:16 in one report (38) and a titer of 1:256 in another report (37), supporting a relationship between serologic activity and follicular involvement. In our study, SA was identified in 23.3% of neonates, which was substantially higher than the 3% reported by Wechselberg and Schneider (35), with a marked male predominance (83.3%). Similar trends have been observed in pediatric acquired syphilis, where Rolotti et al. (3) reported SA in 18.7% of cases, predominantly affecting male infants (66.6%) and presenting with a diffuse or moth-eaten pattern. In the majority of cases, our series corresponded to McCarthy’s “essential” type, characterized by non-scarring moth-eaten alopecia without other visible syphilitic lesions, mirroring patterns described in adult secondary syphilis (20–22). Differences in incidence rates are likely related to population heterogeneity and the fact that the majority of previous studies did not specifically focus on the systematic characterization of alopecia. In addition, SA may be underdiagnosed due to its subtle clinical presentation and the lack of systematic and routine scalp examinations.

**Table 3:** Summary of previous studies on syphilitic alopecia and hair-related findings.

Author (year)	Country	Study design	Population	*N*	Main findings	Limitations
Barlow (1877) (36)	United Kingdom	Case series	Infants with CS	2	First description of alopecia in CS; involvement of the eyebrows and frontal and temporal regions, with no conclusive microscopic hair findings.	Historical descriptive series; lack of modern serological confirmation and absence of alopecia subtype characterization.
Wechselberg and Schneider (1970) (35)	Germany	Retrospective observational study	Infants with manifest CS	127	Describes the frequency and chronology of clinical manifestations; alopecia was infrequent (~3%).	Not focused on alopecia; absence of trichoscopic evaluation.
Reddy et al. (2006) (38)	United States	Case report	Neonate with early CS	1	First modern report of patchy neonatal alopecia associated with congenital syphilis, with elevated non-treponemal titers (RPR 1:16).	Single case; lack of follow-up; no trichoscopic analysis.
Rolotti et al. (2018) (3)	Argentina	Retrospective observational study	Pediatric population with CS and acquired syphilis	7/36	Diffuse alopecia was observed in three infant patients (*n* = 3).	Small number of congenital cases; inability to determine whether alopecia corresponded to congenital or acquired syphilis; absence of trichoscopic evaluation.
Zhang et al. (2019) (37)	China	Case report	Neonate with early congenital syphilis	1	Patchy “moth-eaten” alopecia involving the entire scalp in a neonate with early congenital syphilis and high non-treponemal titers (RPR 1:256), associated with systemic involvement (hepatosplenomegaly and skeletal changes).	Single case report; absence of trichoscopic evaluation; short follow-up.

RPR, rapid plasma reagin test titers; CS, congenital syphilis.

Mucocutaneous alterations were observed in 60% of our neonates, which is consistent with previous literature and reinforces the idea that a considerable proportion of infants with ECS may remain asymptomatic or present only subtle manifestations (1, 2).

Unlike previous studies, our study incorporated a systematic trichoscopic evaluation correlated with neonatal serologic activity. A significant association was found between alopecia and elevated VDRL titers (>1:128 or PP) (*p* = 0.006; OR: 11.9, 95% CI: 1.67–84.50), suggesting that follicular involvement may reflect a higher treponemal load or increased local inflammatory activity induced by *T. pallidum*. Neonates with elevated VDRL titers more frequently exhibited exclamation-mark hairs (*p* < 0.001), coudability hairs (*p* = 0.005), Pohl–Pinkus constrictions (*p* = 0.025), broken hairs (*p* = 0.035), and zig-zag/ angulated hairs (*p* = 0.046). Among these findings, exclamation-mark hairs (OR 52.6) and coudability hairs (OR 23.2) showed the strongest associations, supporting their potential preliminary value as trichoscopic indicators of syphilitic activity; however, the confidence intervals were wide, reflecting the limited sample size and exploratory nature of the analysis.

These findings partly coincide with the trichoscopic patterns described in SA in adults, including broken hairs, zig-zag or angulated hairs, pigtail hairs, vellus and regrowing hairs, along with yellow and black dots, interfollicular scaling, and empty follicles (22, 23, 39). In adults, these features are interpreted as markers of inflammatory follicular lesions and alterations in the hair cycle occurring in the context of secondary syphilis (40).

Nevertheless, it is important to highlight some significant differences when interpreting these findings in newborns. Unlike SA in adults, neonatal hair follicles are physiologically immature, with a predominance of synchronized hair cycles, finer hair shafts, and incomplete follicular differentiation (16). Consequently, several trichoscopic features commonly considered pathological in adults, such as visible vessels, perifollicular scaling, ponytail hairs, vellus hairs, and short redrawing hairs, may also be observed in healthy neonates as part of normal scalp development (31). Furthermore, while SA in adults usually presents with well-defined clinical patterns, such as diffuse or patchy alopecia, neonatal cases often lack obvious alopecic patches and instead exhibit subtle, diffuse trichoscopic alterations (20, 23, 41). This developmental context limits the direct extrapolation of adult trichoscopic criteria to neonates and underscores the need for cautious interpretation. Taken together, these differences highlight that neonatal syphilitic alopecia should not be regarded as a simple phenocopy of adult SA, but rather a distinct entity in which trichoscopic findings must be correlated with clinical presentation, serologic activity, and disease severity to avoid overdiagnosis.

The presence of exclamation-mark hairs, coudability hairs, and Pohl–Pinkus constrictions—findings also described in alopecia areata (AA) (33, 42)—together with the identification of zig-zag hairs, previously described in neonatal *tinea capitis* caused by *Microsporum canis* (32), suggests that the pattern observed in our study may represent a “mixed” trichoscopic phenotype. This constellation of *AA-like* and *tinea capitis–like* features may reflect the structural fragility of the hair shaft secondary to an inflammatory process affecting the particularly fine and delicate neonatal follicles. Nonetheless, these findings alone cannot fully elucidate the underlying etiology.

Altogether, these observations are consistent with the epithelial-tropic and immune-mediated pathogenesis described in histopathological and immunohistochemical studies (8, 9, 43), supporting a folliculotropic inflammatory response as a central mechanism in CS alopecia. Further histologic and molecular investigations in neonatal populations are required to confirm this hypothesis and establish trichoscopic criteria that reliably distinguish early CS from other neonatal hair disorders.

In this context, neonatal trichoscopy emerges as a valuable complementary diagnostic tool, especially in situations where serological interpretation is limited by PP, false negatives in non-treponemal tests, or interference from maternal IgG antibodies, as recognized in the assessment strategies currently recommended by the CDC (26). In our study, two neonates (6.6%) exhibited a PP, and trichoscopic findings contributed to diagnostic reassessment in these cases. Its non-invasive nature, bedside applicability, and ability to provide high-resolution imaging make trichoscopy especially useful in the NICU setting, as it provides timely diagnostic guidance and supports early initiation of empirical treatment while waiting for definitive results.

The strengths of our study include the use of a standardized image acquisition methodology—combining dry and wet examinations with a handheld dermatoscope—and the direct correlation of trichoscopic findings with quantitative serologic titers. Several limitations should be taken into account, including the small sample size and single-center design, the absence of a control group of healthy or non-syphilitic neonates, and the lack of clinical follow-up to evaluate the evolution of the findings. In addition, no formal inter-observer agreement analysis was performed, and molecular or immunohistochemical confirmation of *T. pallidum* in the affected hair follicles was not available.

Future multicenter and longitudinal studies incorporating histological correlation are needed to validate these findings and establish standardized trichoscopic criteria applicable to the neonatal period. Despite these limitations, our results provide a basis for further research using study designs with greater statistical power to demonstrate more solid and definitive associations.

## Data Availability

The raw data supporting the conclusions of this article will be made available by the authors, without undue reservation.

## References

[1] Fang J Partridge E Bautista G Sankaran D. Congenital syphilis epidemiology, prevention, and management in the United States: a 2022 update. Cureus. (2022) 14:e33009. doi: 10.7759/cureus.33009, 36712768 PMC9879571

[2] Leung AKC Leong KF Lam JM. A case of congenital syphilis presenting with unusual skin eruptions. Case Rep Pediatr. (2018) 2018, 1–3. doi: 10.1155/2018/1761454, 29770234 PMC5889854

[3] Rolotti MF Torres Molina L Garone A Rositto A. Pediatric syphilis: a five-year experience in a single Centre. Dermatol Res Skin Care. (2018) 2:12–7.

[4] Whiting C Schwartzman G Khachemoune A. Syphilis in dermatology: recognition and management. Am J Clin Dermatol. (2023) 24:287–97. doi: 10.1007/s40257-022-00755-3, 36689103 PMC9869822

[5] Rochel-Perez EA Martin-Dorantes MA Mendez-Dominguez N. Estimation of the incidence of congenital syphilis in Mexico between 2019 and 2023. Cureus. (2024) 16:e63913. doi: 10.7759/cureus.63913, 39099895 PMC11298237

[6] Zambrano-Alava SN Ruiz-Alava KJ Mina-Ortiz JB Jaime-Mora VA. Sífilis congénita en América Latina: prevalencia, factores de riesgo y complicaciones en la salud materno-fetal. Revista Científica De Salud BIOSANA. (2024) 4:104–9. doi: 10.62305/biosana.v4i4.204

[7] Newton J Silence C Boetes J Cohen BA. Mucocutaneous manifestations of congenital syphilis in the neonate: a review of a surging disease. Pediatr Dermatol. (2023) 40:238–41. doi: 10.1111/pde.15228, 36583308

[8] Hernández-Bel P Unamuno B Sánchez-Carazo JL Febrer I Alegre V. Alopecia sifilítica: presentación de 5 casos y revisión de la literatura. Actas Dermosifiliogr. (2013) 104:512–7. doi: 10.1016/j.ad.2012.02.009, 22749730

[9] Friedli A Chavaz P Harms M. Alopecia syphilitica: report of two cases in Geneva. Dermatology. (2001) 202:376–7. doi: 10.1159/000051688, 11455166

[10] Schlüpen EM Meurer M Schirren CG Baumann L Volkenandt M. Alopecia specifica in secondary syphilis: molecular detection of *Treponema pallidum* in lesional skin. Eur J Dermatol. (1996) 6:19–22.

[11] Rudnicka L Olszewska M Rakowska A Kowalska-Oledzka E Slowinska M Trichoscopy: a new method for diagnosing hair loss 2008. Available online at: https://www.researchgate.net/publication/23134751 (Accessed November 1, 2025). 18664157

[12] Trüeb RM Dias MFRG. A comment on trichoscopy. Int J Trichology. (2018) 10:147–9. doi: 10.4103/ijt.ijt_13_18, 30386072 PMC6192238

[13] Fernández-Domper L Ballesteros-Redondo M Vañó-Galván S. Trichoscopy: an update. Actas Dermosifiliogr. (2023) 114:327–33. doi: 10.1016/j.ad.2022.12.003 36848957

[14] Kinoshita-Ise M Sachdeva M. Update on trichoscopy: integration of the terminology by systematic approach and a proposal of a diagnostic flowchart. J Dermatol. (2022) 49:4–18. doi: 10.1111/1346-8138.16233, 34806223

[15] Dhurat R. Utility of trichoscopy. Indian J. Dermatopathol. Diagn. Dermatol. (2018) 5:89. doi: 10.4103/ijdpdd.ijdpdd_56_18

[16] Contreras-González L Sánchez-Dueñas LE LópezYáñez-Blanco A. Tricología en pediatría 1st. Buenos Aires: Ediciones Journal. (2025). 19–236.

[17] Rakowska A Slowinska M Kowalska-Oledzka E Rudnicka L. Trichoscopy in genetic hair shaft abnormalities. J Dermatol Case Rep. (2008) 2:14–20. doi: 10.3315/jdcr.2008.1009, 21886705 PMC3157768

[18] Cuperus E Bygum A Boeckmann L Bodemer C Bolling MC Caproni M . Proposal for a 6-step approach for differential diagnosis of neonatal erythroderma. J Eur Acad Dermatol Venereol. (2022) 36:973–86. doi: 10.1111/jdv.18043, 35238435 PMC9310754

[19] So N Yip L Orchard D. Paediatric hypotrichosis: a clinical and algorithmic approach to diagnosis. Australas J Dermatol. (2025) 66:e109–19. doi: 10.1111/ajd.14429, 39992008 PMC12062735

[20] Ye Y Zhang X Zhao Y Gong Y Yang J Li H . The clinical and trichoscopic features of syphilitic alopecia. J Dermatol Case Rep. (2014) 8:78–80. doi: 10.3315/jdcr.2014.1176, 25324910 PMC4195505

[21] Piraccini BM Broccoli A Starace M Gaspari V D’Antuono A Dika E . Hair and scalp manifestations in secondary syphilis: epidemiology, clinical features and trichoscopy. Dermatology. (2015) 231:171–6. doi: 10.1159/000431314, 26139324

[22] Sun HY Wong XL Chen MKY Sebaratnam DF. Trichoscopy of syphilitic alopecia: a systematic review. Sex Transm Infect. (2022) 98:539–40. doi: 10.1136/sextrans-2021-055302, 35135846

[23] Pomsoong C Sukanjanapong S Ratanapokasatit Y Suchonwanit P. Epidemiological, clinical, and Trichoscopic features of syphilitic alopecia: a retrospective analysis and systematic review. Front Med (Lausanne). (2022) 9, 1–7. doi: 10.3389/fmed.2022.890206, 35586075 PMC9108265

[24] Glikas MW Day M Toon M. The resurgence of syphilis: a critical public health concern. J Pediatr Health Care. (2025) 39:479–88. doi: 10.1016/j.pedhc.2024.09.003, 40374258

[25] Herrera-Ortiz A López-Gatell H García-Cisneros S Cortés-Ortiz MA Olamendi-Portugal M Hegewisch-Taylor J . Congenital syphilis in Mexico. analysis of national and international standards from the perspective of laboratory diagnosis. Gac Med Mex. (2019) 155:430–8. doi: 10.24875/GMM.M20000328, 32091027

[26] Workowski KA Bolan GA Centers for Disease Control and Prevention Sexually transmitted diseases treatment guidelines, 2015 MMWR Recomm Rep 2015 64 1–137. Available online at: http://www.ncbi.nlm.nih.gov/pubmed/26042815 (Accessed November 3, 2025). PMC5885289 26042815

[27] Pitana T Wolf J Grando A. Prevalência da soropositividade para sífilis congênita na Região Centro-Sul do estado do Rio Grande do Sul. SaBios-Revista Saude Biol. (2022) 17:1–11. doi: 10.54372/sb.2022.v17.3235

[28] Mascareñas De Los Santos AH Castillo Bejarano JI Vaquera Aparicio DN Arcos Viscarra PS Rosales González SP Alvarado Lara DA . Epidemiologic characterization and risk factors for congenital syphilis in Northeast Mexico: a case-control study 2016-2024. Pediatr Infect Dis J. (2025) 44:246–50. doi: 10.1097/INF.0000000000004584, 39733273

[29] Atay Ünal N Savaş Şen Z Güneş Ö Tüfekçioğlu E Yükkaldıran P Kulalı F . Neonatal syphilis: clinical and laboratory assessments and postnatal treatments for infants born to infected mothers. Eur J Clin Microbiol Infect Dis. (2025) 44:1099–105. doi: 10.1007/s10096-025-05073-0, 39971833

[30] Tellería RL Dumondín V Cirio A Buján MM Costa M Buchovsky A . Syphilis in childhood: a retrospective study in a pediatric hospital. Dermatol Argent. (2017) 23:66–72.

[31] Neri I Piccolo V Cocchi G Starace M Patrizi A Dika E . Hair in newborns and infants: clinical and dermoscopic evaluation of 45 cases. Br J Dermatol. (2013) 169:896–900. doi: 10.1111/bjd.12459, 23746094

[32] Zhi HL Xia XJ Shen H Lv WW Zhong Y Sang B . Trichoscopy for early diagnosis and follow-up of pet-related neonatal tinea capitis. Mycopathologia. (2023) 188, 571–575. doi: 10.1007/s11046-023-00709-1, 36652037

[33] Xu L Liu KX Senna MM. A practical approach to the diagnosis and management of hair loss in children and adolescents. Front Med (Lausanne). (2017) 4:112. doi: 10.3389/fmed.2017.00112, 28791288 PMC5522886

[34] Furdon SA Clark DA. Scalp hair characteristics in the newborn infant. Adv Neonatal Care. (2003) 3:286–96. doi: 10.1016/j.adnc.2003.09.005, 14695500

[35] Wechselberg K Schneider JD. Morbidität und klinische Symptomatik der konnatalen Lues im Säuglingsalter. DMW - Deutsche Medizinische Wochenschrift. (1970) 95:1976–81. doi: 10.1055/s-0028-1108765, 5458698

[36] Barlow T. Alopecia in congenital syphilis. Lancet. (1877) 110:276–7. doi: 10.1016/S0140-6736(02)48512-1

[37] Zhang B Wei L Ma L. A neonate with unexplained hair loss. J Pediatr. (2019) 212:240. doi: 10.1016/j.jpeds.2019.04.050, 31130289

[38] Reddy S Bushore D Levy A Skinner RB. Early diffuse alopecia in a neonate with congenital syphilis. Pediatr Dermatol. (2006) 23:564–6. doi: 10.1111/j.1525-1470.2006.00310.x, 17155999

[39] Doche I Hordinsky MK Valente NYS Romiti R Tosti A. Syphilitic alopecia: case reports and trichoscopic findings. Skin Appendage Disord. (2017) 3:222–4. doi: 10.1159/000477415, 29177154 PMC5697519

[40] Tejapira K Sakpuwadol N Pomsoong C Ratanapokasatit Y Suchonwanit P. Trichoscopic features of syphilitic alopecia and alopecia areata: a comparative study. Clin Cosmet Investig Dermatol. (2023) 16:2259–69. doi: 10.2147/CCID.S424054, 37608922 PMC10441631

[41] Mareeva AN Katunin GL Rubtsov AB. Differential diagnostics of syphilitic alopecia and alopecia areata: the clinical picture and trichoscopic signs. Vestn Dermatol Venerol. (2019) 95:34–9. doi: 10.25208/0042-4609-2019-95-3-34-39

[42] Raheem A Al-Dhalimi M. Comparative study of trichoscopic features of alopecia Areata between adults and children and between different body parts (scalp, beard, eyebrow, and moustache). Indian J Dermatol. (2024) 69:285–91. doi: 10.4103/ijd.ijd_346_23, 39296703 PMC11407578

[43] Nam-Cha SH Guhl G Fernández-Peña P Fraga J. Alopecia syphilitica with detection of *Treponema pallidum* in the hair follicle. J Cutan Pathol. (2007) 34 Suppl 1:37–40. doi: 10.1111/j.1600-0560.2006.00726.x, 17997737

